# Effects of Carboxymethyl Chitosan/Pectin Coating Containing Free and Nanoliposome *Mentha piperita* Essential Oil on the Shelf Life of Shrimp During Ice Storage

**DOI:** 10.1002/fsn3.70184

**Published:** 2025-04-22

**Authors:** Ainaz Khodanazary

**Affiliations:** ^1^ Department of Fisheries Faculty of Agriculture and Natural Resources, Gonbad Kavous University Gonbad Kavous Iran

**Keywords:** biopolymers, *Mentha piperita* essential oil, nanoliposome, quality properties, shrimp

## Abstract

The aim of this study is the estimation of the influence of carboxymethyl chitosan (CMCS)/pectin coating containing *Mentha piperita* essential oil (MP EO) nanoliposomes (NLs) on melanosis, sensory analysis, bacterial counts, physicochemical properties, and color analysis of shrimp during iced storage. In addition, the impact of MP EO on the shrimp's polyphenol oxidase (PPO) enzyme inhibition at different concentrations was measured. For this aim, the treated fillets were divided including (1) control, (2) sodium metabisulfite (SMS), (3) MP EO coating, (4) MP EO NLs, (5) MP EO‐CMCS/pectin, and (6) MP EO NLs‐CMCS/pectin coating. Two percentage of MP EO indicated the maximum inhibitory effect of PPO enzyme after 1 and 3 min with values of 75% and 64%, respectively. At the end of storage, the highest and lowest total mesophilic bacteria (TMB) and total psychrotrophic bacteria (TPB) counts were related to control (10.88 and 8.25 log CFU/g) and MP EO NLs‐CMCS/pectin coating (6.87 and 6.90 log CFU/g), respectively. The coated shrimp improved the physicochemical properties (such as total volatile bases‐nitrogen [TVB‐N], pH, peroxide value [POV], and thiobarbituric acid [TBA]) during storage on ice. The lowest rate of TVB‐N (30.33 mg/N100g) and pH (7.06) was recorded in shrimp coated with MP EO NLs‐CMCS/pectin on day 12. During storage for 12 days, the MP EO NLs were better than the other treatments in reducing the rate of oxidation of lipids of shrimp (POV [2.12 meq peroxide/1000 g lipid] and TBA [3.02 mg MDA/kg]). Moreover, shrimp treated with MP EO NLs‐CMCS/pectin had higher odor, texture, color, and overall acceptability scores, compared to the others, after the storage of 12 days at 0°C. Overall, these findings suggest that MP EO NLs‐CMCS/pectin‐based nanocomposite coating could be utilized as an alternative packaging method in seafood products with notable antioxidant, antibacterial, and nutritional properties.

## Graphical abstract

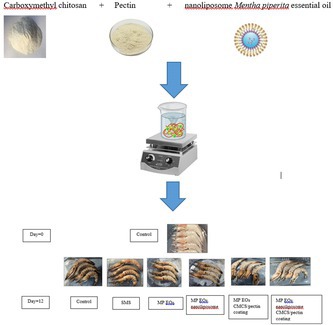

## 1 Introduction

Shrimp is a popular seafood consumed worldwide and enthusiastically welcomed by consumers owing to its flavor and high nutrient content, particularly its abundant protein and unsaturated fatty acids (Wei et al. 2024). Shrimp is very susceptible to lipid oxidation, microbial spoilage, and melanosis during chilled storage, which limits its consumption (Nami et al. 2024). Melanosis, also known as enzymatic browning or black‐spot, is activated by polyphenol oxidase (PPO), which causes the oxidation of phenolic compounds in shrimp into quinones; then they are converted into melanin (high molecular weight dark pigments) under the carapace of the shrimp cephalothorax. The development of the melanosis process depends on storage conditions or external stressors after harvesting shrimp. This discoloration problem is not harmful to customers' health. However, this blackening process decreases the market value of shrimp, leading to drastic economic loss in the shrimp industry. PPO, also known as tyrosinase, polyphenolase, phenoloxidase, phenolase, cresolase, catecholase, and catechol oxidase, has its enzyme activity in the melanosis process inhibited by sulfite‐based compounds and their derivatives in crustaceans, especially shrimp (Sae‐leaw and Benjakul 2019). These chemical materials in consumers show some allergic reaction symptoms. Therefore, using natural preservatives like plant extracts and essential oils (EO) can be a suitable alternative to sulfite compounds to prolong the shelf life of shrimp and prevent melanosis during transportation and processing. Recently, bioactive compounds of EO in different parts of the plant (fruits, seeds, stem, roots, and leaves), includingterpenes, phenols, and aldehydes, have been increasingly used as excellent additives for functional seafood due to biological activities, such as antioxidant and antimicrobial activities (Meenu et al. 2023). Many researchers have widely used EOs to delay the melanosis, bacteriological, and physicochemical spoilage in various shrimp species (Hung et al. 2023; Queiroga et al. 2023). Among EOs, *Mentha piperita* essential oil (MP EO), belonging to the Lamiaceae family, has been found to have high antioxidant and antimicrobial activities (Saharkhiz et al. 2012; El Omari et al. 2024). It is native to Europe and the Middle East, but it can now be found in the USA, China, India, the former USSR, Hungary, France, Iran, and Italy (Gholamipourfard et al. 2021). It has been proven that EOs are used restrictively because of the interaction between volatile oxidation compounds (Bakkali et al. 2008); environmental factors such as light, oxygen, and high temperatures; and their taste (Sharma et al. 2021). Thus, it is recommended that EO is not used directly to preserve foods. On the other hand, EOs have hydrophobic nature and poor water solubility in polar solutions. To be efficient, EOs can be coated with a hydrophilic substances to improve their performance (Azizi et al. 2024). Recently, novel processing technology was introduced to extend the shelf life of seafood enriched with EOs for better quality. Encapsulation technology can protect EOs against chemical degradation of active compounds. Liposomes, as phospholipid‐based surfactants, are formed with spherical vesicles with a bilayer membrane shaped by the hydrophobic tails joining together (Mozafari 2010). The encapsulation process for producing carriers or nanoparticles is called nanoliposomes (NLs). NLs can protect biologically active substances like essential oils, enzymes, vitamins, and antimicrobials against environmental stresses and control their release over a specific period of time and under specific conditions (Wu et al. 2015). Also, EO‐loaded NLs are frequently used in food packaging due to lower sensory effects. NLs are one of the types of nano‐carriers that can transport both hydrophilic and hydrophobic materials simultaneously with the flexible properties of nanoliposomes, either inside a membrane of a vesicle or bilayer. Several studies reflect the beneficial effects of nanoliposomes compared with liposomes, such as greater controlled release, better solubilization, greater surface area, and more effective targeting of encapsulated compounds (Anvar et al. 2023; Haghju et al. 2016; Zomorodian et al. 2023).

The use of active packaging, as an important new technology, for the protection of seafood in the form of biodegradable films and edible coatings has attracted a lot of attention (Vásconez et al. 2009). Based on good functional properties, such as mechanical properties and gas barrier properties, polysaccharides are widely used in food packaging (Xiong et al. 2020). To prepare an edible coating or film with higher efficiency, adding EOs as antioxidant and antimicrobial materials is the most common ingredient to delay the oxidation of lipids, ensure safety, and increase the quality of seafood because biopolymers are good candidates for carrying EOs (Azevedo et al. 2018; Azizi et al. 2024; Saghari et al. 2024). Among polysaccharide biopolymers, chitosan and pectin are the common compounds for forming bi‐layer edible/biodegradable coatings with higher performance (Esmaeili and Khodanazary 2021). Pectin is derived from a plant cell wall and is applied to make active packaging due to its nontoxic, biocompatible, inexpensive natural polymer, and good barrier property against oxygen (Espitia et al. 2014; Moslemi 2021). Despite the positive effect of pectin, it has poor moisture barrier properties and nonantioxidant and nonantibacterial activities (Asdagh et al. 2019; Šešija et al. 2018). Combining pectin with other biopolymers can improve its antioxidant and antibacterial properties. Inclusion of pectin with chitosan can be an effective strategy to improve the inherent shortcomings. The polysaccharide‐based chitosan is obtained from the deacetylation of chitin found widely in the shells of crustaceans and fungi (Elsabee and Abdou 2013). The potential applications of chitosan are limited because of its solubility in acidic liquids (Yang et al. 2023). Carboxymethyl chitosan (CMCS), as a derivative of chitosan, is obtained from the carboxymethylation of chitosan. Compared with chitosan, CMCS remarkably improves some properties such as strong solubility over a broad pH range, mucoadhesive characteristics, and absorption enhancement (Shariatinia 2018). On the other hand, CMCS retains the advantageous biological properties of chitosan such as antioxidant and antimicrobial properties (Sun et al. 2020). The mechanism of the interaction between pectin and CMCS is explained by electrostatic interactions between the negatively charged pectin (COO^−^) and the positively charged side‐chain groups in CMCS (NH^2+^). Therefore, the application of pectin and CMCS in packaging materials formulations has gained much attention. In export industries of crustaceans, especially shrimp, one important problem is the prevention of melanosis. In addition, no study has been found on the usage of MP EO on PPO inhibition and shrimp shelf life extension. A little published data exist on the associated usage of nanoliposome and biopolymers in combination with natural compounds for the evaluation of seafood quality (Nami et al. 2024; Abdollahzadeh et al. 2023; Azizi et al. 2024). So far, no research has been done regarding the preservation of shrimp using the EO of *Mentha piperita* (free and nanoliposome form) in Iran and other countries, and on the other hand, due to the high consumption of shrimp in Iran and the world, doing this research seems necessary. Therefore, the aim of the present study is to investigate the comparative effect of the free forms and nanoliposome of MP EO with CMCS/pectin coating to increase the shelf life of shrimp ( *Metapenaeus affinis* ) using PPO inhibition, PPO enzyme activity, melanosis, sensory properties (odor, texture, color, and overall acceptability), bacterial analysis (TMB and TPB), physicochemical tests (TVB‐N, pH, TBA, and POV), and color analysis.

## 2 Materials and Methods

### 2.1 Materials

Lecithin (soybean phospholipid; PubChem CID: 5287971) and pectin were obtained from Sigma–Aldrich, Germany. Sodium metabisulfite (PubChem CID: 656671) and Carboxymethyl cellulose were obtained from Tetra‐Chem (Ingersoll, Canada) and Avijeh Farjood parsi Co., Iran, respectively. Peppermint EO was bought from Barij Essence Pharmaceutical Company, which was confirmed by Iranian pharmacognosy specialists. Other solvents and reagents were of analytical grade or higher available purity.

### 2.2 Preparation of Nanoliposomes/MP EO Nanoliposome/CMCS/Pectin Solution of MP EO Nanoliposome

For producing nanoliposomes, 5 g of lecithin was mixed with 95 mL of distilled water for preparing 5% (w/v) lecithin. Subsequently, the suspension was vortex‐mixed at room temperature for 30 min to achieve dispersion. Subsequently, the suspension underwent sonication for 180 s at 40 kHz and 40% power (1 s on and 1 s off) (UHP‐400, Topsonics Sonicator, Iran), resulting in a colloidal suspension. An ice bath was used to prevent sample heating during the ultrasound application. Nanoliposomes were kept in dark bottles at 4°C. For preparing MP EO NLs, 2 g of MP EO was dissolved in 100 mL of lecithin solution and then was sonicated for 180 s at 40 kHz. Finally, for the formation of CMCS/pectin coating of MP EO NLs, 20 mL of MP EO‐NLs were mixed with 80 mL of CMCS (Avijeh Farjood parsi Co., Iran)/pectin (Sigma–Aldrich, Germany) solution (containing 1% w/v of CMCS and 2% w/v of pectin) (v/v) for 48 h in the dark.

### 2.3 Preparation of Shrimp Samples and Coating of Shrimp With Various Prepared Nanovesicles

Freshly caught shrimp *Metapenaeus affinis* were collected from the Persian Gulf in Khuzestan province, Iran. The average weight of shrimp was 16.12 ± 0.02 g per shrimp. Immediately after collection, samples were cooled with ice and transported to the seafood‐processing laboratory. The ratio of shrimp to ice was 1:3 (w/w). Upon arrival, shrimp were washed in cold water. Afterward, shrimp samples were randomly assigned into six treatment lots consisting of: (1) control (covered by distilled water), (2) sodium metabisulfite (SMS) (preparing by dissolving 1.25 g SMS in 100 mL of deionized water), (3) MP EO coating (dissolving 2 g of MP EO in 100 mL of deionized water), (4) MP EO NLs, (5) CMCS/pectin coating‐MP EO, and (6) MP EO‐NLs‐CMCS/pectin coating. All treatments were immersed at a shrimp‐to‐solution ratio of 1:2 (w/v) at 4°C for 1 min. Then, the shrimps were removed and allowed to drain for 5 min at ambient temperature (20°C) in order to form the edible coating. All samples were sealed in polyethylene bags (three samples for each treatment), maintained at 0°C for 12 days in insulated boxes with a shrimp‐to‐ice ratio of 1:2 (w/w) to evaluate at five different days (0, 3, 6, 9, and 12 days).

### 2.4 Preparation of PPO From Shrimp

The process described by Nirmal and Benjakul (2009) was used to extract polyphenol oxidase (PPO) enzyme. For this purpose, the cephalothoraxe of several shrimps were separated and then ground with liquid nitrogen in a mixer. The powder (50 g) was mixed with 150 mL of the extracting buffer (0.05 M sodium phosphate buffer, pH 7.2, containing 1.0 M NaCl, and 0.2% Brij‐35). The mixture was stirred continuously at 4°C for 30 min, followed by centrifugation at 8000 *g* at 4°C for 30 min using a refrigerated centrifuge (5425R, Eppendorf, Germany). Solid ammonium sulfate was added into the supernatant to obtain 40% saturation and allowed to stand at 4°C for 30 min. The precipitate was collected by centrifugation at 12,500 *g* at 4°C for 30 min using a refrigerated centrifuge. The pellet obtained was dissolved in a minimum volume of 0.05 M sodium phosphate buffer, pH 7.2, and dialyzed against 15 vol of the same buffer at 4°C with three changes of dialysis buffer. The insoluble materials were removed by centrifugation at 3000 *g* at 4°C for 30 min and the supernatant was used as “crude PPO extract.”

#### 2.4.1 PPO Performance Evaluation

L‐DOPA was used as a substrate to evaluate PPO activity. For this purpose, the approach presented by Nirmal and Benjakul (2009) was used with some modifications. The assay included crude PPO (0.1 mL), 0.05 M sodium phosphate (0.4 mL), 15 mM L‐DOPA (0.6 mL) in deionized water, pH 6.0, and deionized water (0.1 mL). We assessed PPO activity at 45°C for 3 min via monitoring the dopachrome formation at 475 nm. For this purpose, a spectrophotometer was used (UV Biochrom, Libra S12, UK). In order to produce the enzyme and substrate blanks, the enzyme and substrate were removed from the test tube, filled with distilled water. PPO functional unit included an absorbance rise of 0.001 at 475 nm/min per mL.

### 2.5 Inhibitory Effect of MP EO on PPO Activity

Various concentrations of 100 μL MP EO (1%, 2%, 3%, and 4% *w*/*v*) were combined with crude PPO (100 μL) to achieve 0.5%, 1%, 1.5%, and 2% final concentrations (*w*/*v*). The mixture was incubated for 30 min at 37°C. Afterward, we added assay buffer (0.05 M sodium phosphate, 0.4 mL), and the reaction was initiated by adding 15 mM L‐DOPA (0.6 mL) (at 45°C). The reaction was conducted at 45°C, and we measured the absorbance for 3 min at 475 nm. The same procedure was applied for preparing the control, but MP EO was used instead of deionized water. After determining the impact of MP EO on PPO activity, the inhibitory activity was evaluated and presented as the inhibition percentage: (%) Inhibition = (A − B)/A × 100 with A representing PPO control activity and B denoting the activity of PPO when MP EO is present.

### 2.6 Browning Function Assessment

The browning of white‐leg shrimp was evaluated by 12 panelists. A 10‐point scale based on the International Organization for Standardization standard ISO 8586‐1 (2023) was used for this purpose. The authors asked panel members to give a browning score between 0 and 10 to the shrimp. A score of 0 showed lack of browning, 2 represented limited browning that affects up to 20% of the surface of the shrimp. A score of 4 denoted average browning (20%–40% of surface of shrimp), 6 showed significant browning (40%–60% of the surface of shrimp), 8 implied severe browning (60%–80% of the surface of shrimp), and 10 showed very severe browning (covering more than 80% up to the entire surface). For assessing the browning, samples were taken every 4 days in a 12‐day period for each treatment.

### 2.7 Sensory Assessment

Six trained members applied the process described by Wu (2014) in order to perform the sensory assessment of samples. They evaluated the appearance, odor, overall acceptability, color, and texture of shrimp samples using organoleptic assessment. Rating of each parameter was done individually on a descriptive hedonic scale from 1 to 9. Overall acceptability was rated from 9 (totally acceptable) to 1 (totally unacceptable), smell ranged from 9 (highly pleasant) to 1 (smell of spoilage), texture was rated from 9 (sold and firm) to 1 (very soft), and color from 9 (shiny and bright) to 1 (matte and dark). A score of 5 was considered the critical acceptance threshold for each feature, and ratings below that indicated rejection of desired sensory features. The environmental conditions of the sensory assessment included ambient light, room temperature with a relative humidity of 45%, and natural ventilation.

### 2.8 Bacterial Analysis

After mixing shrimp meat sample (1 g) and homogenizing it with physiological serum solution (9 mL), the necessary dilutions (10^−1^—10^−6^) were developed (ICMSF 1986). One milliliter of dilutions was employed for bacterial culturing. Plate agar counting (PCA) was used to determine total mesophilic bacteria (TMB) and total psychrotrophic bacteria (TPB) of shrimp through the spreading plate approach following incubation of plates for 2 days at 35°C and for 7 days at 4°C, respectively (ICMSF 1986).

### 2.9 Physicochemical Test

The total volatile bases nitrogen (TVB‐N) content was measured by the microdiffusion method. The microdiffusion method was determined by distillation after the addition of MgO to a homogenized fish sample. The distillate was collected in a flask containing an aqueous solution of boric acid and methyl red as an indicator. Afterward, the boric acid solution was titrated with sulfuric acid (H_2_SO_4_) solution. The TVB‐N value (mg N/100 g of fish) was determined according to the consumption of sulfuric acid (Goulas and Kontominas 2005).

The thiobarbituric acid (TBA) content was analyzed due to Siripatrawan and Noipha's (2012) method with some modification. Ten grams of homogenized sample was added with 97.5 mL of distillated water and 2.5 mL of 4 M HCl. The mixture was heated with steam distillation. Five milliliters of distillate was added to 5 mL of thiobarbituric reactive reagent containing 0.02 M TBA in 90% glacial acetic acid and incubated in boiling water for 35 min. After cooling, the absorbance of the pink solution was measured at 532 nm using a spectrophotometer. The TBA value is expressed as mg malonaldehyde equivalents/kg sample.

The measurement of pH was carried out on 5 g of sample homogenized in 45 mL distilled water. The pH value of the sample was determined using a digital pH meter (913 pH meter, Metrohm, Herisaw, Switzerland) (Suvanich et al. 2000).

The peroxide value (POV) content of the fillets was measured in the lipid extract following the method of Woyewoda et al. (1986). Results were expressed in meq peroxide/1000 g lipid (Woyewoda et al. 1986).

### 2.10 Measurement of Shrimp Shell Color

An automatic colorimeter was used to measure shrimp color, and the procedure described by Zhang et al. (2015) was employed. It ranged from 0 (dark) to 100 (white); L* (light) denotes vividness. The spreading of the b* (yellowness) scale is from negative to positive values for yellow, and a* (redness) covers negative to positive values for green. Three parts of the shrimp (body, tail, and head) were used for assessing shell color. For each sample in every section of the shell, three measurements were taken, and the average values were registered.

## 3 Statistical Analysis

All experiments were performed at least in triplicate and shown as the mean ± standard error. The results were subjected to one‐way analysis of variance and independent samples *t*‐tests using SPSS 24 statistical software (SPSS Inc. Chicago, IL, USA). *p <* 0.05 was considered to be statistically significant.

## 4 Results and Discussion

### 4.1 Compounds Identification of *M. piperita* EO

The chemical composition of *M. piperita* EO included menthol (55.25%), menthone (23.22%), menthyl acetate (7.36%), 1,8‐cineole (5.91%), caryophyllene (5.27%), β‐pinene (1.01%), α‐pinene (0.78%), β‐bourbonene (0.29%), α‐cubebene (0.14%), and longipinene (0.04%).

### 4.2 Changes in Activity and Inhibition of PPO

**FIGURE 1:**
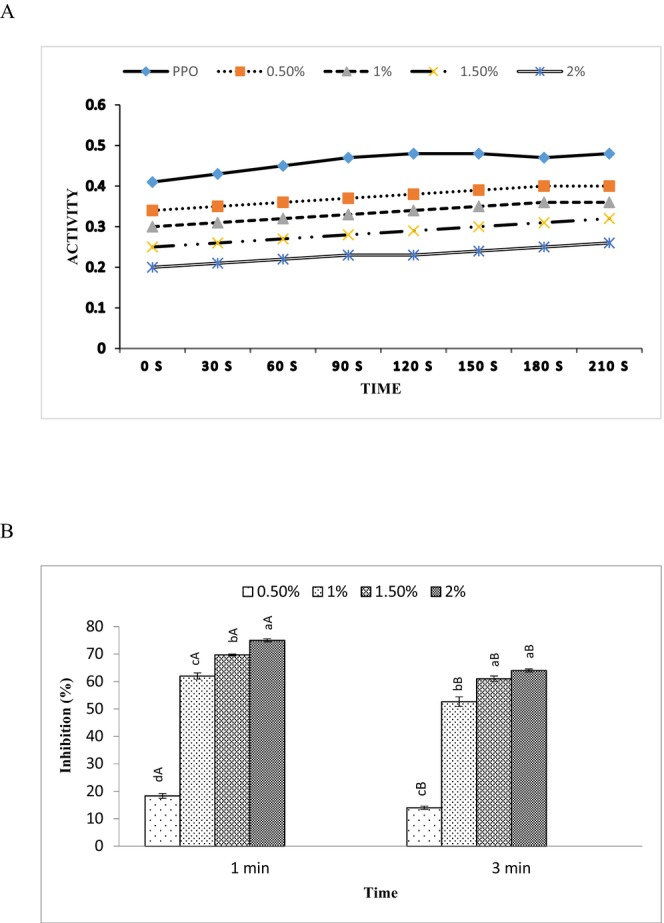
Effect of different concentrations of MP EO (*Mentha piperita* essential oil) at the time on PPO (polyphenol oxidase) activity (A). The inhibitory effect of MP EO on PPO activity (B). Mean values and standard errors from the three replicates are presented. The different capital letters within the same concentration indicate the significant differences (*p* < 0.05). The different small letters within the same time indicate the significant differences (*p* < 0.05).

Figure 1A,B illustrates the inhibitory effects of MP EO on PPO activity and inhibition of PPO with different concentrations of MP EO (0.5%, 1%, 1.5%, and 2%) until 210 s. The different concentrations of MP EO showed PPO inhibition activity. With increasing concentration used of MP EO, the PPO activity decreased with a reduction in absorbance value, exhibiting enhancement of the inhibition of PPO by MP EO. Although the PPO activity of 2% MP EO‐treated shrimp was lower in comparison to other concentrations of MP EO during the whole storage period (*p <* 0.05). As observed, the results showed that the best effect of 2% MP EO treatment on PPO activity and inhibition of PPO of shrimp was determined (*p <* 0.05). It is known that different EOs have potent inhibitory activity against the diphenolase activity of tyrosinase (Chang et al. 2013). MP EO has some active compounds such as phenols, polyphenols, terpenoids, alkaloids, lectins, and polypeptides (Rezvani et al. 2019; Taherpour et al. 2017). These compounds inhibited PPO activity by interacting with the active sites of the enzymes via hydrogen bond/or hydrophobic interaction and also by reduction of quinone formed due to competition of MP EO and L‐DOPA for the enzyme binding site (Sae‐leaw and Benjakul 2019). Furthermore, polyphenol and its derivatives could act as the primary category of inhibitors of tyrosinase (polyphenoloxidase) and also suppress PPO activity by binding with the active site of the enzyme (Kubo and Kinst‐Hori 1998). Additionally, it has been reported that polyphenols have reducing power (reduction of quinone) and metal chelating capacity. Phenolic compounds act as chelating agents of Cu^2+^ in the catalytic site of PPO for decreasing the activity of PPO because the active site of PPO consists of two copper atoms (Kubo and Kinst‐Hori 1998). The above results indicate that the hydroxyl group of the active components of MP EO might inhibit the PPO activity due to reduction of DOPA‐chrome to DOPA. This finding is consistent with the results reported by Sae‐leaw et al. (2017), who indicated that catechin and its derivatives significantly inhibited PPO activity in Pacific white shrimp. In another study, Zhang et al. (2024) reported that polyvinyl alcohol/chitosan film incorporated with cinnamon essential oil has shown the best inhibitory effect on PPO of Pacific white shrimp.

### 4.3 Changes in Melanosis

**FIGURE 2:**
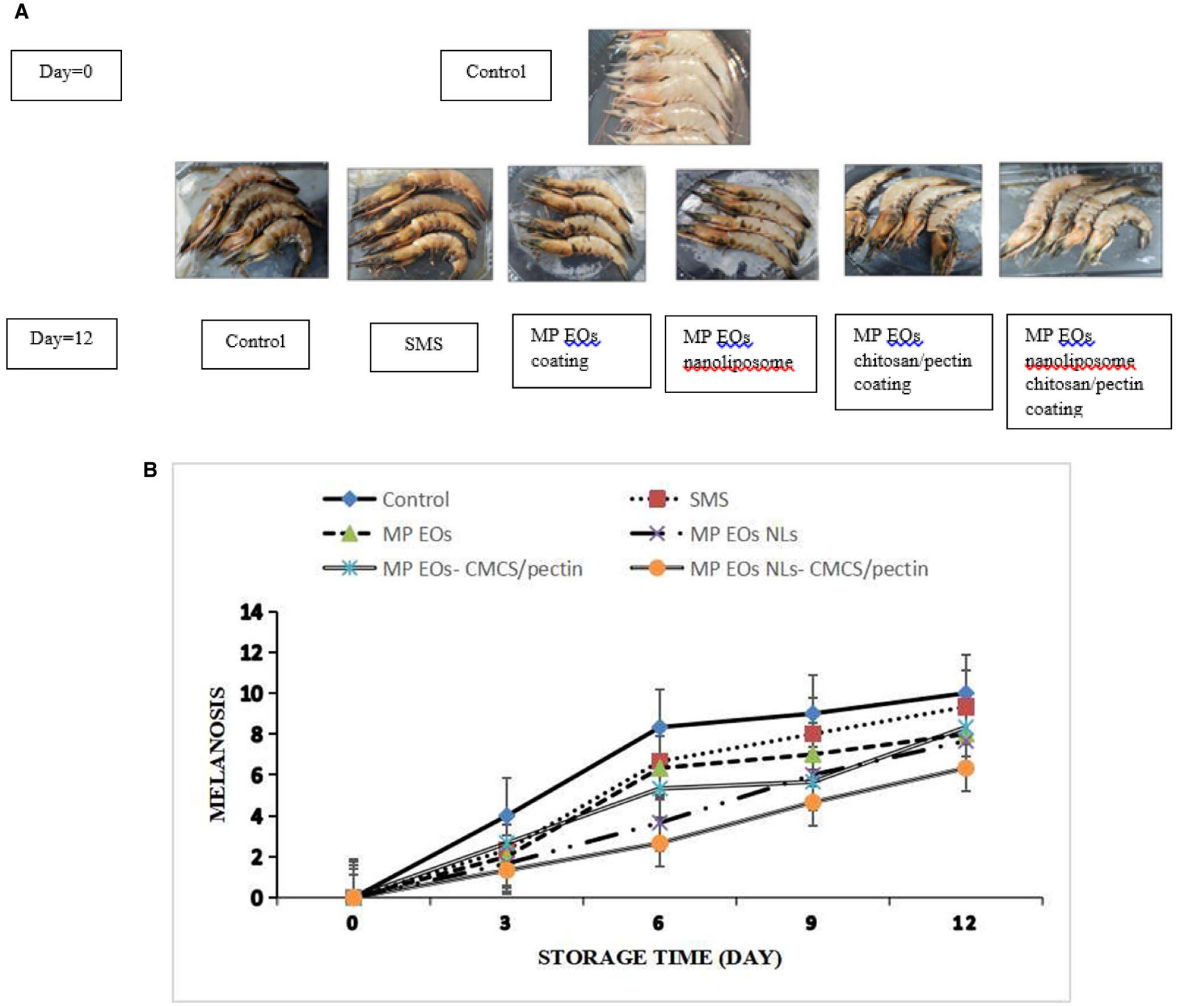
Changes in appearance of shrimp with different treatments during ice storage (A). Melanosis score of shrimp with different treatments during ice storage (B). Mean values and standard errors from the three replicates are presented. The different capital letters within the same storage time indicate the significant differences (*p* < 0.05). The different small letters within the same treatment indicate the significant differences (*p* < 0.05). MP EO‐ CMCS/pectin, *Mentha piperita* essential oil‐carboxymethyl cellulose/pectin; MP EO NLs‐CMCS/pectin, *Mentha piperita* essential oil nanoliposome‐ carboxymethyl cellulose/pectin; MP EOs NLs, *Mentha piperita* essential oil nanoliposome; MP EOs, *Mentha piperita* essential oil; SMS = sodium metabisulfite.

The changes in appearance and melanosis scores of shrimp with different treatments during ice storage are shown in Figure 2A,B. At the beginning of storage, all samples had no melanosis (score = 0). Melanosis increased in all samples when the storage time increased (*p* < 0.05); however, it varied between groups. In the control sample, melanosis was evident on the third day. The melanosis of the control sample was slow in the first 3 days, then rapidly increased, and finally showed more melanosis than other groups. The SMS‐treated shrimp had a significantly lower melanosis score compared to control (*p* < 0.05) during the first 6 days of storage. This study proved that SMS could prevent the formation of lack spot with intermediate quinone or forming sulfoquinone (Ferrer et al. 1989). No difference in melanosis score was noticeable in samples treated with MP EO or SMS throughout the storage of 12 days (*p* > 0.05). From day 9 onward, the melanosis scores of MP EO NL and MP EO‐CMCS/pectin coating were similar (*p* > 0.05). Generally, during 12 days of storage, the MP EO‐CMCS/pectin coating‐treated shrimp had the least melanosis formation, followed by MP EO nanoliposome, MP EO CMCS/pectin coating, and then MP EO. It is possible that the main compounds in MP EO, such as polyphenol, inhibit PPO activity due to chelating copper atoms of the active site in PPO through the hydroxyl substitution in the rings and reduce the amount of melanosis in shrimp during storage. On the other hand, PPO is synthesized by zymogen (prepolyphenol polyoxidase) in crustaceans, which is activated by cell wall proteases such as lipopolysaccharidase, peptidoglycanase, and 1,3‐b‐glucanase (Nirmal and Benjakul 2011). Therefore, polyphenol compounds with antimicrobial properties could inhibit the activities of bacteria, in which PPO could be inhibited effectively and reduce melanosis in crustaceans. Many studies have reported that plant polyphenols have an antimelanogenesis effect in crustaceans, especially shrimp, during storage (Basiri et al. 2015; Sae‐leaw and Benjakul 2019). The shrimp treated with MP EO NL reinforced inhibition of PPO, leading to retardation of melanosis. On the other hand, CMCS/pectin coating might impact the PPO activity by forming a biodegradable coating as a barrier against oxygen transfer. Therefore, MP EO NL‐CMCS/pectin coating could effectively reduce the melanosis of white shrimp during iced storage.

### 4.4 Sensory Analysis

**FIGURE 3:**
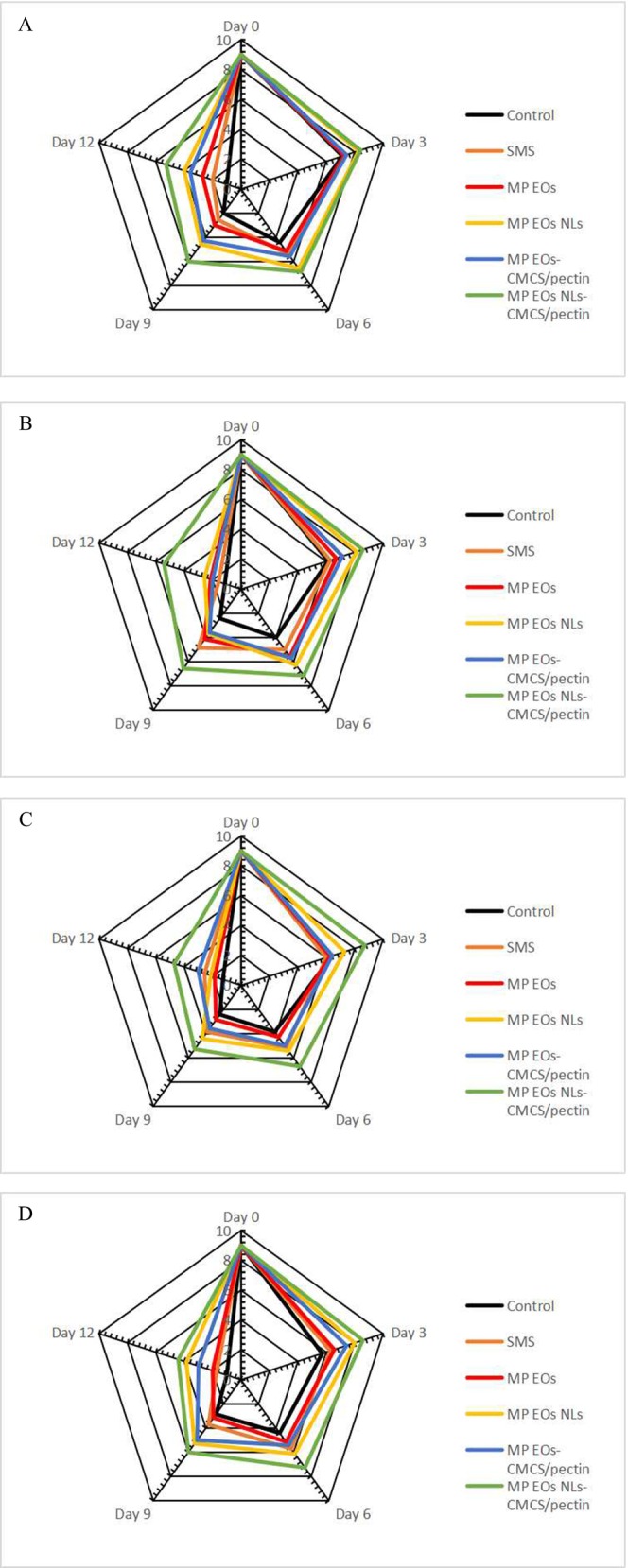
Sensory analysis (including: Odor (A), texture (B), color (C), and overall acceptability (D)) of shrimp with different treatments during ice storage. Mean values and standard errors from the three replicates are presented. The different capital letters within the same storage time indicate the significant differences (*p* < 0.05). The different small letters within the same treatment indicate the significant differences (*p* < 0.05). MP EO NLs‐CMCS/pectin, *Mentha piperita* essential oil nanoliposome‐carboxymethyl cellulose/pectin; MP EO‐CMCS/pectin, *Mentha piperita* essential oil‐carboxymethyl cellulose/pectin; MP EOs NLs, *Mentha piperita* essential oil nanoliposome; MP EOs, *Mentha piperita* essential oil; SMS, sodium metabisulfite.

Figure 3A–D shows changes in the likeness scores of the uncoated and treated groups during storage on ice. At day 0, the likeness scores of all samples were similar. The initial sensory properties, includingodor, texture, color, and overall acceptability, of all samples were 9, indicating that the untreated and treated white shrimp were of high acceptability. Due to putrid odor, mushy texture, off flavor, and lack of complete acceptance, shrimp with scores lower than 5 were regarded as unsuitable. Bacterial activity and protein breakdown in flesh form some gases such as hydrogen sulfide and ammonia, leading to spoilage and an unacceptable odor in shrimp (Peng et al. 2022). With increasing storage time, likeness scores diminished after 12 days of storage (*p* < 0.05). At the end of storage, the likeness scores were found in descending order: control, SMS, MP EO, MP EO‐CMCS/pectin sample, MP EO NL, MP EO NL‐CMCS/pectin sample, respectively. The results showed that polyphenol compounds in the MP EO sample improved the sensory attributes. Nanoliposome of MP EO had better sensory properties compared to the samples coated with free MP EO on the storage time of shrimp, which efficiently prohibited the growth of bacteria in samples and reduced the rate of protein degradation as well as the accumulation of volatile compounds in shrimp. Additionally, MP EO NLs played a major role in the improvement of sensory scores, with prevention of lipid oxidation (Saghari et al. 2024). Overall, it is concluded that the MP EO NLs could increase sensory quality and the amount of nutrients, especially polyunsaturated fatty acids (PUFA) of shrimp during ice storage due to control of lipid oxidation with a reduction in the production of free radical formation and toxic oxidation products (Nami et al. 2024). On the other hand, EO NLs could decrease the rate of protein degradation and the accumulation of volatile compounds in shrimp (Saghari et al. 2024). It is worth noting that free fatty acids (FFAs) produced during the fat hydrolysis process lead to a decrease in the stability of proteins by reacting with FFAs and proteins, which causes tissue destruction (Hematyar et al. 2019). Nami et al. (2024) showed that soy protein isolate combined with phycocyanin‐loaded nanoliposomes had a better effect on the sensory properties of white shrimp. The results reconfirmed that nanoliposome and biopolymer coating showed a positive impact on the acceptability or likeness of shrimp meat. Moreover, Afifi et al. (2023) also reported that nanocomposite coating (pullulan‐nano clay) activated with nanoliposomes containing watercress essential oil improved sensory properties. Over time, shrimp coated with MP EO NLs‐CMCS/pectin were still acceptable on the 12th day. These results justify that CMCS/pectin coating reinforced with MP EO NLsprolonged the shelf life of shrimp.

### 4.5 Changes in Bacterial Counts

**FIGURE 4:**
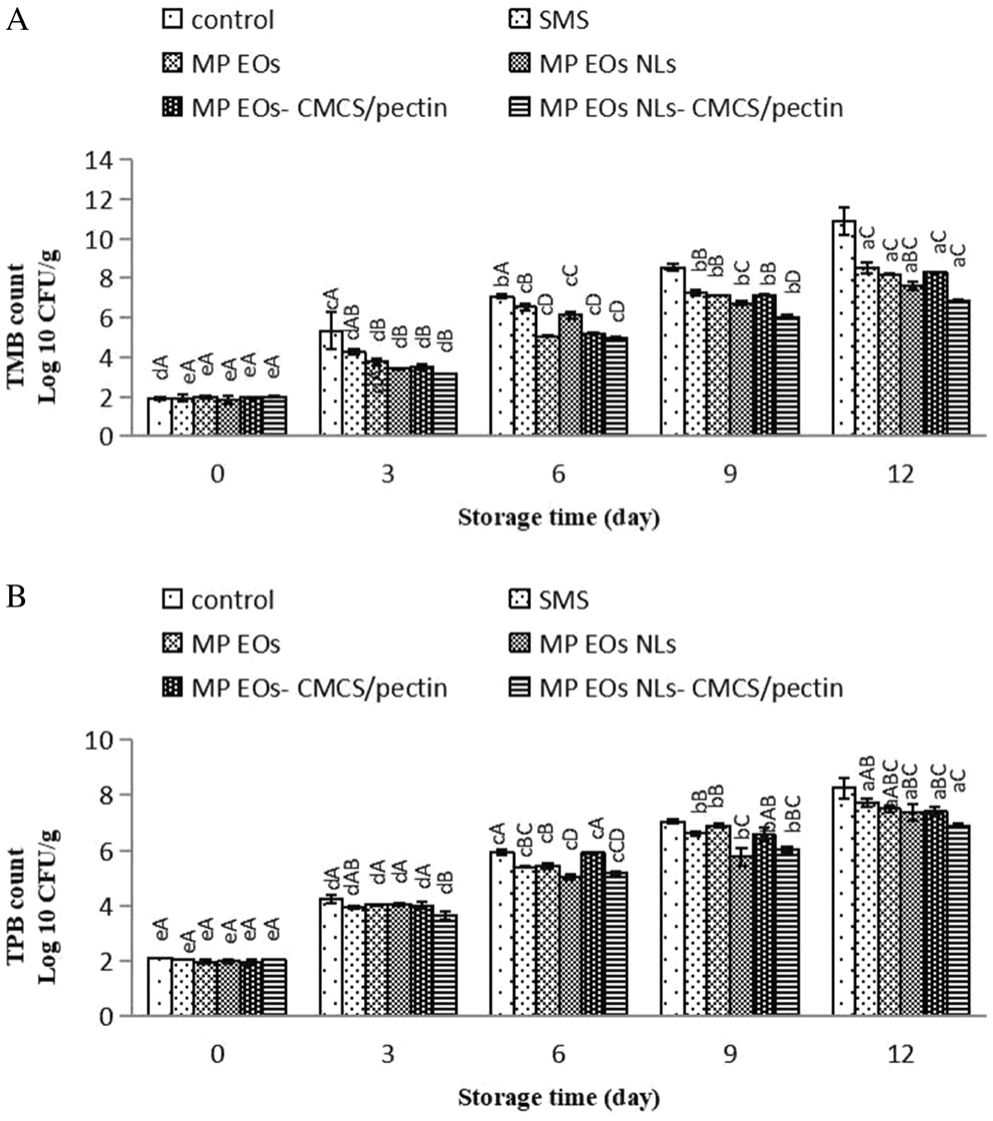
Changes in TMB (total mesophilic bacteria) (A) and TPB (total psychrotrophic bacteria) (B) count of shrimp during ice storage. Mean values and standard errors from the three replicates are presented. The different capital letters within the same storage time indicate the significant differences (*p* < 0.05). The different small letters within the same treatment indicate the significant differences (*p* < 0.05). MP EO NLs‐CMCS/pectin, *Mentha piperita* essential oil nanoliposome‐carboxymethyl cellulose/pectin; MP EO‐CMCS/pectin, *Mentha piperita* essential oil‐carboxymethyl cellulose/pectin; MP EOs NLs, *Mentha piperita* essential oil nanoliposome; MP EOs, *Mentha piperita* essential oil; SMS, sodium metabisulfite.

Protein, as one of the most abundant compounds in seafood, was decomposed by microorganisms and enzymatic action (Singh et al. 2016). The bacterial quality (total mesophilic bacteria (TMB) and total psychrotrophic bacteria (TPB)) of different treatments during ice storage in comparison with the control is depicted in Figure 4A,B. The initial TMB (log_10_ CFU/g) in the control was 1.92 log_10_ CFU/g; it was 1.94, 1.99, 1.84, 1.95, and 2.00 log_10_ CFU/g for SMS, MP EO, MP EO NLs, MP EO‐CMCS/pectin sample, and MP EO NLs‐CMCS/pectin sample, respectively, showing a high baseline quality of the shrimp. Moreover, the shrimp's initial TPB count ranged from 1.96 to 2.13 log_10_ CFU/g in all samples. As observed, the result of bacteria at day 0 agrees with that reported by Nami et al. (2024). TMB and TPB in all treated shrimps exhibited an increasing trend during the storage time. The results showed that SMS has decelerated the bacterial population in comparison with the control samples. The increasing rate of TMB and TPB in samples treated with nanoliposome was slower than in samples treated with free EO, indicating that the samples coated with the MP EO with/or without CMCS/pectin had very little effect on decreasing the bacterial growth trend. There are many researches about the reduction of bacteria count in coated shrimp by using different biopolymers combined with essential oil (Mehraie et al. 2023; Liu et al. 2020). Previous studies had shown that phenolic and flavonoid compounds of plant EOs were associated with antibacterial activity against different food‐borne bacteria (Olmedo et al. 2013; Chaudhari et al. 2021; Baptista et al. 2020). According to Fincheira et al. (2023), MP EO consists of different bioactive compounds including α‐terpinene, isomenthone, transcarveol, pipertitinone oxide, and β‐caryophyllene, leading to strong antimicrobial properties in food products (Saharkhiz et al. 2012). Antimicrobial activity of MP EO was mentioned by Gholamipourfard et al. (2021). The antibacterial mechanisms of EO can be explained by (1) interacting bioactive components present in EO with the phospholipid bilayer of the bacterial membrane, which disturbed the structural integrity of the lipopolysaccharide layer of the outer membrane of bacteria and then influenced the cell metabolism causing cell death; (2) interacting EO with hydrolytic enzymes, cell envelope transport proteins, and carbohydrates on the bacterial cell surface; (3) inhibiting nucleic acid synthesis; and (4) metal chelating property of EO against a range of bacteria (Burt 2004; Cowan 1999; Rodriguez et al. 2016). As observed, the lowest TMB and TPB counts (6.87 ± 0.06 and 6.90 ± 0.07 log_10_ CFU/g, respectively) belonged to the MP EO NLs‐CMCS/pectin sample on the 12th day; nanoliposome and biopolymers could increase the antibacterial properties. Abdollahzadeh et al. (2023) found the total viable counts and total pseudomonads count of rainbow trout fillets coated with nanochitosan/nano‐encapsulated essential oil of Golpar (*Heracleum persicum* L.) were the lowest count (6.8 and 8 log CFU/g, respectively) at the end of storage time. These results seem to be quite consistent with the results of the antibacterial activity assay that confirmed the high antibacterial potential of EO NLs and biopolymers compared to other coatings (Azizi et al. 2024; Abdollahzadeh et al. 2023). The presence of MP EO NL and CMCS/pectin in samples had a stronger effect as the most significant reduction in the bacteria counts. MP EO, when used as nanoliposome, strengthens the antimicrobial properties of CMCS‐pectin‐based coating. It is probably due to the encapsulation of bioactive compounds in the nanoliposome for greater protection of the compounds from degradation for a longer period and their easier transfer to the cell wall of bacteria (Shabani et al. 2023). Also, nanoliposome helps decrease the MP EO evaporation. Nanoliposome of EO significantly intensified the microbial inhibitory effect due to the interaction of nanoliposomes with bacterial cells (Azizi et al. 2024). The bacterial growth mechanism prohibition of nanoliposome has been attributed to the cell type, their higher surface area and closer proximity of nanodroplets with the cells of bacteria, and the physicochemical properties of the liposome membrane. Nanoliposomes facilitate the release of active ingredients of the core material (EO) inside the bacterial cell because of improving the surface‐to‐volume ratio of particles at the nanoscale, significantly intensifying the microbial inhibitory effect (Wu et al. 2015). Similar studies were performed for meat, shrimp, and fish fillet by Sayyari et al. (2021); Nami et al. (2024); Shabani et al. (2023). The CMCS‐pectin acts as a barrier against oxygen transfer. Moreover, the addition of bioactive compounds of EO to CMCS‐pectin‐coating solutions resulted in improved antibacterial properties of these coatings by interacting with biopolymers and reducing the movement of antimicrobial agents into foods (Sharma et al. 2021). The combination of EO with CMCS‐pectin coating acts as a barrier against the transmission of oxygen, strengthening the synergistic action between natural products and biopolymers. The permissible limit of TMB is 6 log CFU/g for fresh shrimp (Liao et al. 2018). By day 6 of storage, TMB counts in shrimp reached about 6 log_10_ CFU/g (7.66 log_10_ CFU/g) for control samples, which is higher than the acceptable levels in raw shrimp, showing a microbiological shelf life of about 5–6 days for the control samples. Samples coated with SMS, MP EO, and MP EO NLs were suitable for eating for up to 9, 9, and 12 days of storage, respectively. TMB counts for MP EO NLs‐CMCS/pectin did not surpass the allowable limits at the end of the storage period. In summary, the results of the synergistic action of composite coating with MP EO NL demonstrated the potential of MP EO NL to enhance the antimicrobial activity of the composite coating.

### 4.6 Changes in Physicochemical Properties

#### 4.6.1 Physicochemical Changes

##### 4.6.1.1 TVBN Value

**FIGURE 5:**
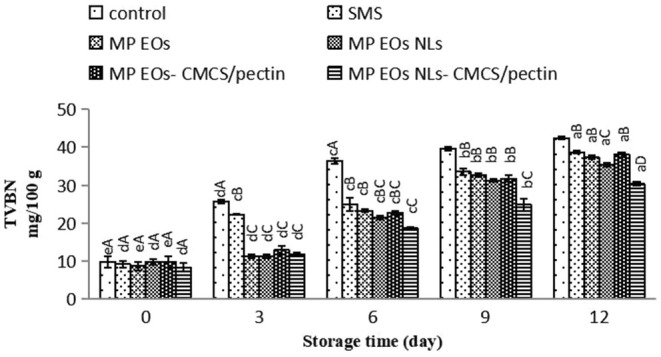
Changes in TVBN (total volatile bases nitrogen) of shrimp during ice storage. Mean values and standard errors from the three replicates are presented. The different capital letters within the same storage time indicate the significant differences (*p* < 0.05). The different small letters within the same treatment indicate the significant differences (*p* < 0.05). MP EO NLs‐CMCS/pectin, *Mentha piperita* essential oil nanoliposome‐carboxymethyl cellulose/pectin; MP EO‐CMCS/pectin, *Mentha piperita* essential oil‐ carboxymethyl cellulose/pectin; MP EOs NLs, *Mentha piperita* essential oil nanoliposome; MP EOs, *Mentha piperita* essential oil; SMS, sodium metabisulfite.

The TVB‐N is an index indicator to evaluate raw shrimp's quality (Alizadeh‐Sani et al. 2020). Figure 5 illustrates the changes in TVB‐N contents of the control sample and different treatments during the entire storage time. The primary TVB‐N value in all samples ranged between 8.40 and 9.80 mg N/100 g of flesh. The TVB‐N values of the control and treated samples increased slightly with prolonging the storage time (*p* < 0.05) due to the deamination of free amino acids, oxidation of amines, degradation of nucleotides, and activity of spoilage microorganisms such as *Pseudomonas*, H_2_S‐producing bacteria, and Enterobacterales of shrimp (Tometri et al. 2020; Sae‐leaw and Benjakul 2019). As revealed in this figure, the changes in TVB‐N content in various treatments were different due to their various protective activities. The TVB‐N value of shrimp coated with MP EO NLs was much lower than shrimp treated with MP EO (*p* < 0.05). There was no difference between SMS and MP EO (*p* > 0.05). The lowest TVB‐N content was observed in the MP EO NLs‐CMCS/pectin sample, suggesting MP EO NLs‐CMCS/pectin is most effective in decreasing the degradation of nitrogenous compounds of shrimp. This treatment may have helped retain the biological properties of nanoliposome and biopolymers. In a similar study, Azizi et al. (2024) stated the TVB‐N value of rainbow trout fillet coated with whey protein containing nanoliposome dill ( *Anethum graveolens* L.) essential oil was the lowest value (22.5 mg N/100 g) after 21 days of storage. This study shows that the increase in TVB‐N content over time was in agreement with the reduction of bacterial load during storage, which may be due to the activity of bacterial species producing alkaline metabolites from the multiplying bacteria or the excretion of proteins. Na et al. (2018) showed a direct relationship between TVB‐N content and microbial growth in Pacific white shrimp. Generally, polyphenolic compounds could combine with adenosine monophosphate deaminase, which decreased the formation of volatile compounds like H_2_S, histamine, ammonia, and foul‐smelling trimethylamine in shrimp (Sae‐leaw and Benjakul 2019; Mehraie et al. 2023). A level of 30 mg N/100 g is the acceptable level of TVB‐N in fresh aquatic products according to the China National Food Safety Standard Methods (GB/T 5009.228–2016). Therefore, the best storage periods for shrimp treated with SMS, MP EO, MP EO NLs, and MP EO + CMCS/pectin were 6 days, while the control sample reached this limit on day 3 of the storage period. In this study, TVB‐N values of MP EO NLs/CMCS‐pectin remained below this limit of acceptability throughout the entire storage. It is likely caused by delaying bacterial spoilage and endogenous enzyme activities.

##### 4.6.1.2 pH Value

**FIGURE 6:**
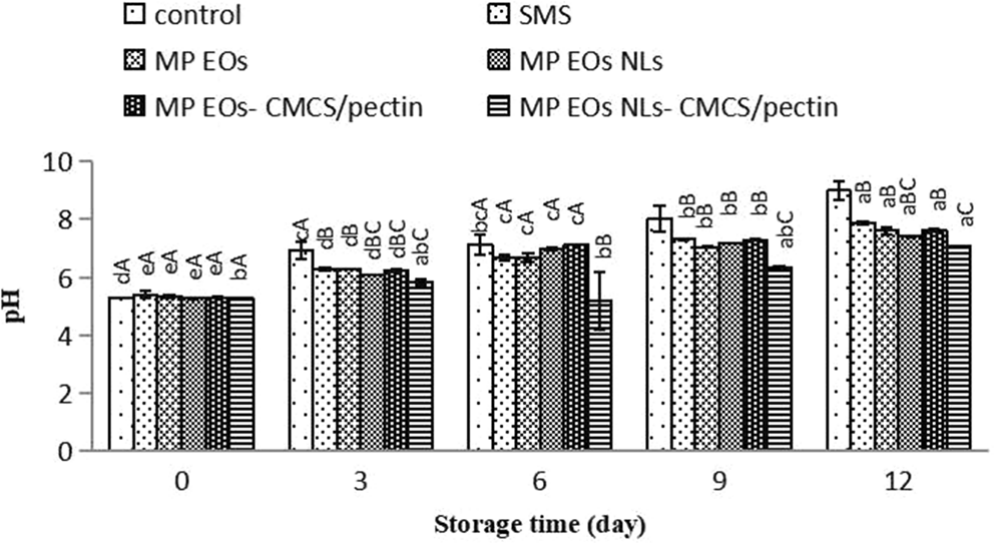
Changes in pH of shrimp during ice storage. Mean values and standard errors from the three replicates are presented. The different capital letters within the same storage time indicate the significant differences (*p* < 0.05). The different small letters within the same treatment indicate the significant differences (*p* < 0.05). MP EO NLs‐CMCS/pectin, *Mentha piperita* essential oil nanoliposome‐carboxymethyl cellulose/pectin; MP EO‐CMCS/pectin, *Mentha piperita* essential oil‐carboxymethyl cellulose/pectin; MP EOs NLs, *Mentha piperita* essential oil nanoliposome; MP EOs, *Mentha piperita* essential oil; SMS, sodium metabisulfite.

Figure 6 shows the changes in pH value for shrimp samples during cold storage. On day 0, the pH content in different treated samples ranged between 5.30 and 6.03. The pH content significantly increased when storage time increased up to 12 days (*p* < 0.05). pH content reached to 8.66 in the control‐, 7.60 in the SMS‐, 7.77 in the MP EO‐, in the MP EO NLs, MP EO‐CMCS/pectin‐ and 7.04 in the MP EO NLs‐CMCS/pectin‐treated samples at the end of the storage period. It is probably because of (1) activity of bacteria enzymes for degradation of proteins leading to a raise in alkaline substances such as ammonia, tri‐methylamine, and volatile basic compounds; (2) autolysis process by endogenous enzymes (proteases and lipases) for decomposition of fish protein and lipids under sufficient oxygen; (3) production of the alkaline materials (such as indole, histamine, ammonia, and trimethylamine); (4) decarboxylation of amino acids leading to the formation of amines (Nirmal and Benjakul 2011; Shabani et al. 2023; Kılıç et al. 2014). The pH value had a direct relationship with bacterial load and TVBN content. Based on our findings, the pH content in the control samples was considerably higher than in the other treated samples. Among all of the treatments, MP EO NLs/CMCS‐pectin‐treated samples had the lowest pH value during storage. It may be associated with the inhibitory effect of phenolic compounds and biodegradable coatings on bacterial enzyme activity and carbon dioxide permeability produced by microbial activity during the storage period. According to Osanloo et al. (2023), EO‐containing samples were decreased bacterial growth and later degradation of amino complexes due to antibacterial activity of phenolic compounds. Compared with the free MP EO, shrimps coated with MP EO nanoliposome showed lower values of pH (*p* < 0.05) due to their strong effect of antibacterial properties. These results support previous findings on synergistic action between essential oil with nanoliposome form and biopolymers for retardation in increasing the pH content.

##### 4.6.1.3 TBA and POV Values

**FIGURE 7:**
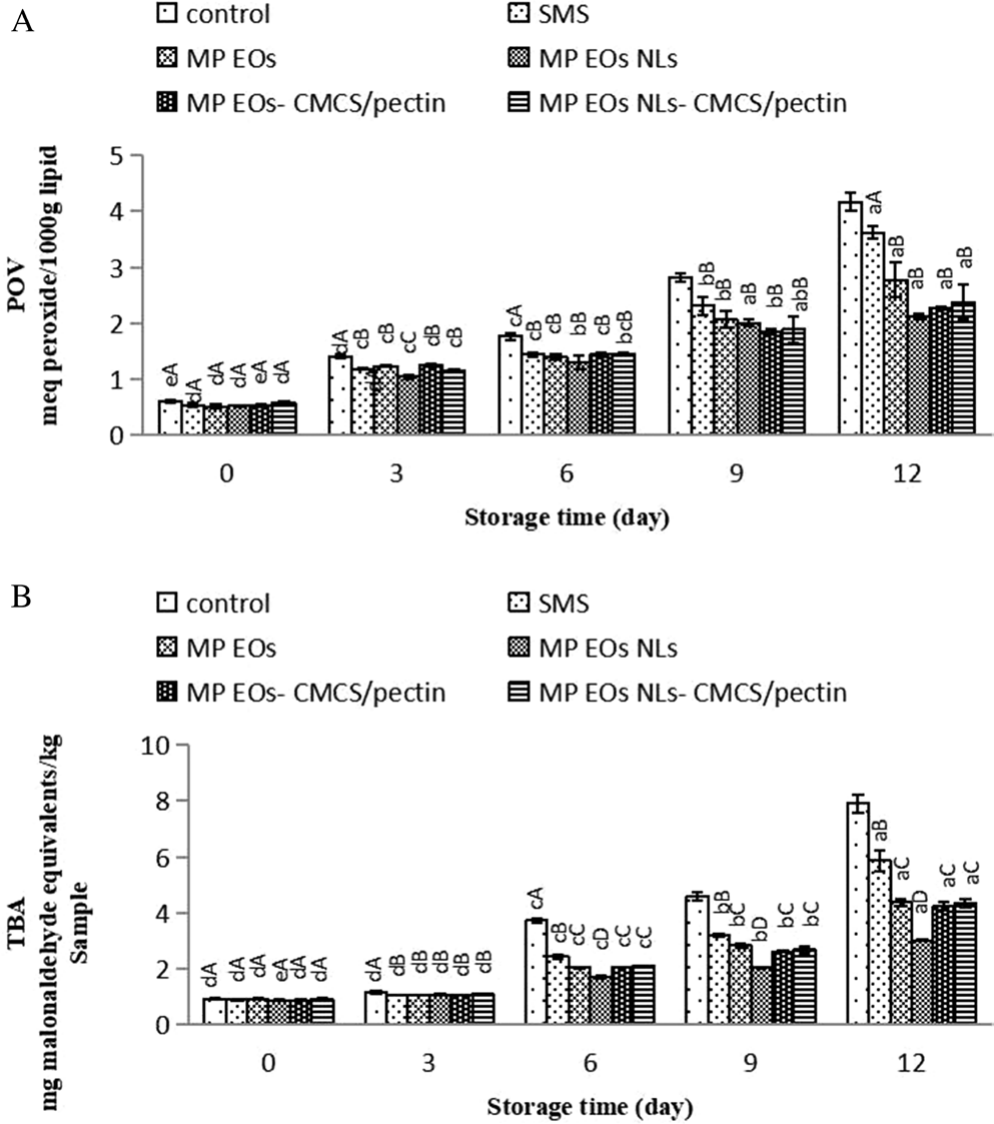
Changes in POV (peroxide value) (A) and TBA (thioarituric acid) (B) count of shrimp during ice storage. Mean values and standard errors from the three replicates are presented. The different capital letters within the same storage time indicate the significant differences (*p* < 0.05). The different small letters within the same treatment indicate the significant differences (*p* < 0.05). MP EO NLs‐CMCS/pectin, *Mentha piperita* essential oil nanoliposome‐carboxymethyl cellulose/pectin; MP EO‐CMCS/pectin, *Mentha piperita* essential oil‐carboxymethyl cellulose/pectin; MP EOs NLs, *Mentha piperita* essential oil nanoliposome; MP EOs, *Mentha piperita* essential oil; SMS, sodium metabisulfite.

The peroxide value (POV) index, as a first indicator of lipid oxidation, measures the concentration of hydroperoxide during the oxidation process of fatty acids and also the free radicals formed due to eliminating the fatty acids double bond and reacting with oxygen (Chaijan 2011; Homayonpour et al. 2021). Variations in the POV content of shrimp during ice storage are shown in Figure 7. At day 0, the POV content ranged from 0.04 to 0.06 meq peroxide/1000 g lipid. The POV value in control and treated samples was raised during the 12‐day storage. On the 16th day, the highest POV content was for control samples (1.94 meq peroxide/1000 g lipid). The MP EO NLs/CMCS‐pectin coated fillet slowed the production of POV during storage, which means that MP EO NLs and CMCS‐pectin could reduce the lipid oxidation of fillets. It is worth noting that the enhancement of nanoliposome efficiency value with the CMCS‐pectin coating might be attributed to the EO nanoliposome and biopolymers' capacities to decrease lipid oxidation to form peroxides/hydroperoxides. Azizi et al. (2024) stated that whey protein coating combined with EO of dill ( *Anethum graveolens* L.) nanoliposomes compared to the other treatments had better results on POV (11.5 mEq/Kg). Moreover, Homayonpour et al. (2021) reported that sardine fillets treated with nano‐chitosan coating incorporating with free/nanoencapsulated cumin EO decreased POV during storage through limiting the oxidation of lipids.

**FIGURE 8:**
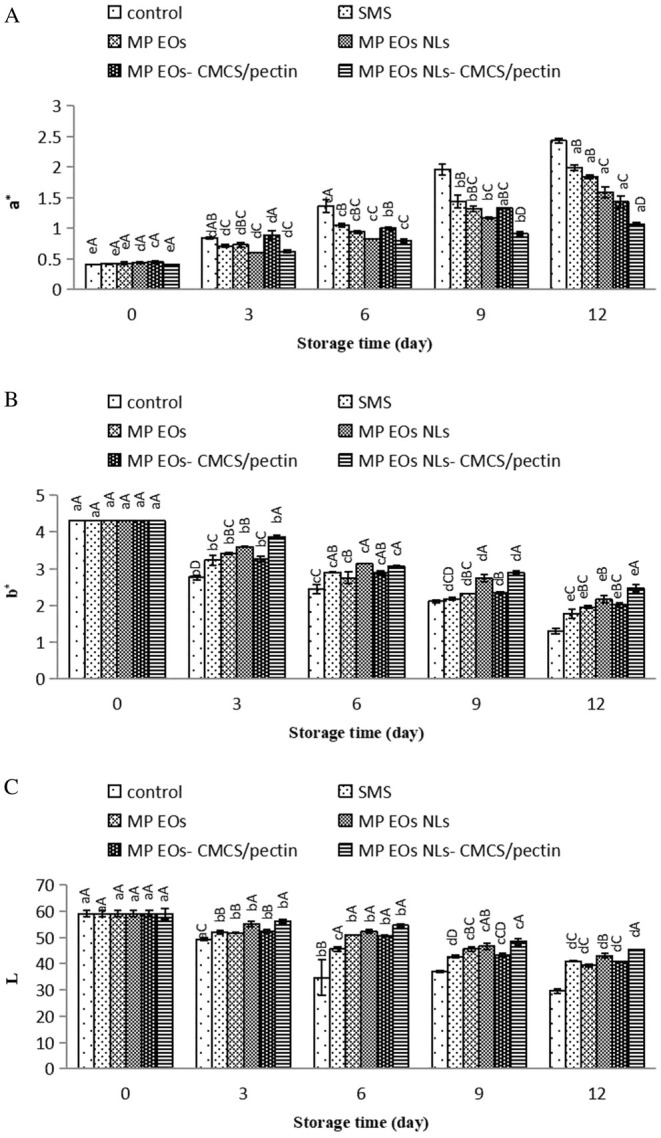
Changes in color (a* (A), b* (B), and *L* (C)) of shrimp during ice storage. Mean values and standard errors from the three replicates are presented. The different capital letters within the same storage time indicate the significant differences (*p* < 0.05). The different small letters within the same treatment indicate the significant differences (*p* < 0.05). MP EO NLs‐CMCS/pectin, *Mentha piperita* essential oil nanoliposome‐carboxymethyl cellulose/pectin; MP EO‐CMCS/pectin, *Mentha piperita* essential oil‐carboxymethyl cellulose/pectin; MP EOs NLs, *Mentha piperita* essential oil nanoliposome; MP EOs, *Mentha piperita* essential oil; SMS, sodium metabisulfite.

The TBA test, as an essential index of lipid oxidation, measures malondialdehyde (MDA) produced due to the oxidation of unsaturated fatty acids (Heydari‐Majd et al. 2019). The changes in TBA values of all the samples are stated in Figure 8. The initial TBA values of all samples were 0.59–0.84 mg MDA/kg, indicating the freshness of the shrimp. This result is in agreement with the results found by Basiri et al. (2015). The TBA value of the control and SMS remarkably increased during ice storage (*p* < 0.05). As observed, the TBA value of the SMS sample was higher than the control sample (*p* < 0.05). The increase in TBA content indicates lipid rancidity of seafood, which leads to the production of an unpleasant smell and taste and shortens shelf life (Alsaggaf et al. 2017). The MP EO‐treated shrimp had lower malondialdehyde (MDA) values, compared to SMS with storage (*p* < 0.05) because of the slow release of EO on the surface of shrimp. Also, the antioxidant mechanism of MP EO might be through the strong antioxidant activity with radical scavenging capacities with the antioxidant agent due to the high oxygenated monoterpene content, mainly 1,8‐cineole, rotundifolone, menthofuran, pulegone, and menthol (Gholamipourfard et al. 2021). At the end of the storage period, the highest and lowest TBA content were obtained in the control and samples treated with MP EO NLs, respectively (*p* < 0.05). According to our findings, the TBA value of MP EO NLs remained constant, while the MP EO‐CMCS/pectin, MP EO, and MP EO NL‐MCS/pectin indicated a slight increase during storage. It should be noted that the antioxidant effects of MP EO, MP EO‐CMCS/pectin, and MP EO NL‐MCS/pectin were the same. It was noted that MP EO NL had a better effect on TBA content, indicating that the antioxidant activity of EO NLs was better than EO free or EO (free or nanoliposome) combined with biopolymers. These results suggest that interactions among EO and CMCS/pectin to immobilize EO and prevent free interaction with oxidizing free radicals could lead to reduced antioxidant activity of the MP EO NL‐CMCS/pectin. Remarkably, pure CMCS has insufficient antioxidant activity (Xu et al. 2021). According to Patsias et al. (2006), the maximum level of TBA value is 5 mg malonaldehyde/kg as the acceptable limit of fresh seafood products. Based on this study, coated samples with the MP EO NLs had lower contents than the recommended level at the end of the storage period, which indicated the good quality of shrimp regarding lipid oxidation during storage on ice. However, TBA values in control samples, SMS, MP EO, MP EO‐CMCS/pectin, and MP EO NL‐MCS/pectin were not acceptable levels. Therefore, it can be concluded that the NLs could preserve the EO from degradation and evaporation, and also a high specific surface area of EO could accelerate their antioxidant activity.

#### 4.6.2 Change in Color

The color is a main attribute of seafood products for acceptability and preference by consumers (Soysal et al. 2015). Color parameters of the shrimp including lightness (L*) coordinate, redness (a*) value, and blueness (b*) value during ice storage for 12 days are shown in Figure 8A–C. Results indicated that the color properties of shrimp were significantly influenced by using CMCS/pectin coating and MP EO NLs, whereas SMS had no effect on the color parameters. It was also evident that SMS played a substantial role in the color deterioration of shrimp. L* and b* values of treated shrimps significantly decreased over the storage period (*p <* 0.05). On the 12th day of storage, the CMCS/pectin coating containing MP EO NLs showed a relatively higher value of b* compared to other treatments. The control sample was greener than the sample containing NLs, which underlines the importance of the NLs on masking the undesirable sensory effects. As seen in the L* values results, CMCS/pectin coating containing MP EO NLs indicated the lowest color change. However, a* of all samples notably increased during the storage period (*p <* 0.05), which indicates an increase in red color in the samples. The rate of increase in a* was lower in the shrimp coated with MP EO NL‐CMCS/pectin in comparison to other samples, which means the muscle color tended to be yellow and red gradually. Similar results were found that NLs could improve the color value of shrimp (Li et al. 2022). Color stability in shrimp during storage might be attributed to slowing down the protein denaturation and then reduction of water evaporation according to the processing procedure (Nakamura et al. 2011). In addition, nonenzymatic browning, proteolysis process by bacterial proteases, and lipid oxidation can directly affect the color deterioration of meat (Cardoso et al. 2016). The color analysis had a significant interaction between packaging material and storage time. The obtained results were in agreement with Chen et al. (2024); Amjadia et al. (2020), who investigated the plant EO NLs on the color of meat.

## 5 Conclusions

The results demonstrate that the combination of MP EO NL with CMCS/pectin solution is an active nanocomposite coating with antimicrobial and antioxidant effects. There was a significant effect between treatments in the control of microbial population. The simultaneous use of CMCS‐pectin and MP EO NL has a high protective effect against bacterial activity of shrimp because of the antagonistic interactions between MP EO NL and CMCS‐pectin, which maintained a nearly constant TVBN and pH during storage. Although this study showed that MP EO and MP EO‐CMCS/pectin had the same effect in killing bacterial growth. MP EO NL displayed good antioxidant properties since POV and TBA values of these samples were lower than those of other samples over the storage period. The findings showed that the addition of NLs to CMCS/pectin solution led to darker and more reddish colors compared to the control samples during long‐term storage. This study was another confirmation of the biological activities of MP EO NLs and CMCS/pectin composite coatings in the seafood packaging industry. One of the limitations of our study was the financial constraints in evaluating and comparing the effect of *Mentha piperita* extract (in the form of aqueous and ethanolic) on shrimp characteristics, which is suggested to be investigated in future research.

## Author Contributions

Ainaz Khodanazary: Investigation, Methodology, Conceptualization, Writing – review and editing.

## Data Availability

The author confirm that data generated or analyzed supporting the findings of this study are available within the article, and any further supporting material will be provided on request from the corresponding author.
